# Actigraphy-Derived Sleep Profiles of Children with and without Attention-Deficit/Hyperactivity Disorder (ADHD) over Two Weeks—Comparison, Precursor Symptoms, and the Chronotype

**DOI:** 10.3390/brainsci11121564

**Published:** 2021-11-27

**Authors:** Mirjam Ziegler, Anna Kaiser, Christine Igel, Julia Geissler, Konstantin Mechler, Nathalie E. Holz, Katja Becker, Manfred Döpfner, Marcel Romanos, Daniel Brandeis, Sarah Hohmann, Sabina Millenet, Tobias Banaschewski

**Affiliations:** 1 Department of Child and Adolescent Psychiatry and Psychotherapy, Central Institute of Mental Health, Medical Faculty Mannheim, Heidelberg University, 68159 Mannheim, Germany; anna.kaiser@zi-mannheim.de (A.K.); christine.igel@zi-mannheim.de (C.I.); konstantin.mechler@zi-mannheim.de (K.M.); Nathalie.Holz@zi-mannheim.de (N.E.H.); daniel.brandeis@zi-mannheim.de (D.B.); Sarah.Hohmann@zi-mannheim.de (S.H.); Sabina.Millenet@zi-mannheim.de (S.M.); tobias.banaschewski@zi-mannheim.de (T.B.); 2 Department of Child and Adolescent Psychiatry, Psychosomatics and Psychotherapy, University Hospital of Würzburg, University of Würzburg, 97080 Würzburg, Germany; Geissler_J@ukw.de (J.G.); romanos_m@ukw.de (M.R.); 3 Donders Center for Brain, Cognition and Behavior, Radboud University Nijmegen, 6525 EN Nijmegen, The Netherlands; 4 Department for Cognitive Neuroscience, Radboud University Medical Center Nijmegen, 6525 EN Nijmegen, The Netherlands; 5 Department of Child and Adolescent Psychiatry, Psychosomatics and Psychotherapy, Medical Faculty, Philipps-University Marburg and University Hospital Marburg, 35039 Marburg, Germany; Katja.Becker@med.uni-marburg.de; 6 Center for Mind, Brain and Behavior (CMBB), University of Marburg and Justus Liebig University Giessen, 35032 Marburg, Germany; 7 Department of Child and Adolescent Psychiatry, Psychosomatics and Psychotherapy, Faculty of Medicine and University Hospital Cologne, University of Cologne, 50931 Cologne, Germany; manfred.doepfner@uk-koeln.de; 8 Department of Child and Adolescent Psychiatry and Psychotherapy, University Hospital of Psychiatry, University of Zürich, 8032 Zürich, Switzerland; 9 Center for Integrative Human Physiology, University of Zürich, 8057 Zürich, Switzerland; 10 Neuroscience Center Zürich, Swiss Federal Institute of Technology, University of Zürich, 8057 Zürich, Switzerland

**Keywords:** sleep, actigraphy, attention-deficit/hyperactivity disorder (ADHD), intra-individual variability (IIV), chronotype, children, continuous performance task (CPT), precursor symptoms, ESCAlife

## Abstract

Although sleep problems are common in children with ADHD, their extent, preceding risk factors, and the association between neurocognitive performance and neurobiological processes in sleep and ADHD, are still largely unknown. We examined sleep variables in school-aged children with ADHD, addressing their intra-individual variability (IIV) and considering potential precursor symptoms as well as the chronotype. Additionally, in a subgroup of our sample, we investigated associations with neurobehavioral functioning (*n* = 44). A total of 57 children (6–12 years) with (*n* = 24) and without ADHD (*n* = 33) were recruited in one center of the large ESCAlife study to wear actigraphs for two weeks. Actigraphy-derived dependent variables, including IIV, were analyzed using linear mixed models in order to find differences between the groups. A stepwise regression model was used to investigate neuropsychological function. Overall, children with ADHD showed longer sleep onset latency (SOL), higher IIV in SOL, more movements during sleep, lower sleep efficiency, and a slightly larger sleep deficit on school days compared with free days. No group differences were observed for chronotype or sleep onset time. Sleep problems in infancy predicted later SOL and the total number of movements during sleep in children with and without ADHD. No additional effect of sleep problems, beyond ADHD symptom severity, on neuropsychological functioning was found. This study highlights the importance of screening children with ADHD for current and early childhood sleep disturbances in order to prevent long-term sleep problems and offer individualized treatments. Future studies with larger sample sizes should examine possible biological markers to improve our understanding of the underlying mechanisms.

## 1. Introduction

ADHD is a neurodevelopmental disorder characterized by age-inappropriate hyperactivity, inattention, and impulsivity, with a high prevalence of about 5% in childhood and adolescence [1]. *The Diagnostic and Statistical Manual of Mental Disorders 5th edition* (DSM-5) [2] distinguishes between the inattentive presentation, the hyperactive/impulsive, and the combined presentation. Sleep problems are not listed in the diagnostic criteria of ADHD but are reported frequently. Estimates assume that up to 50–70% of children, adolescents, and adults with ADHD are affected by sleep disturbances [3,4,5,6], which range from sleep onset insomnia through sleep-disordered breathing, and restless legs syndrome to sleep epilepsy [7,8]. Nevertheless, findings on ADHD and sleep are very heterogeneous regarding the ADHD patients affected with sleep problems (subtype, severity), the specific kind of sleep aberrations, and the relationship between ADHD (dimensions) and sleep problems [9,10].

Within the two-process model of sleep by Borbély [11], the general sleep process is determined by the interplay between the circadian timing (Process C), which reflects the ‘biological clock’ and fluctuates with a nearly 24 h cycle, and the perceived sleep pressure (Process S). A delay in circadian timing leads to drowsiness the next day and accumulates over the days. The most reliable measure of circadian timing is the onset of melatonin secretion in dim light conditions (dim light melatonin onset (DLMO) [12,13]), resulting in the so-called ‘chronotype’ [14]. Normally, children are earlier chronotypes, become later with the start of the puberty, and are ‘latest’ at around the age of 20. With increasing age in adulthood, the chronotype becomes earlier once again [14]. Despite external influences due to social constraints, the chronotype is also genetically determined, with a heritability of about 50% [15]. Questionnaires like the Munich ChronoType Questionnaire (MCTQ [14]) have proven to be a good correlate of DLMO, with the advantage of being easy to administer [16].

Hypotheses about the etiology of sleep problems in ADHD, or the underlying mechanisms, are various and range from a causative role of sleep for overt ADHD symptoms, to a shared genetic or neurofunctional overlap [9,17,18]. Additionally, disturbances in the dopaminergic system play a crucial role in the etiology of ADHD as well as in sleep regulation [9]. In this regard, early regulatory problems like sleep or feeding deviations and excessive crying in young childhood are among the early precursor symptoms of ADHD and are linked to gene polymorphism of the dopaminergic system [19,20,21]. Future behavior problems might be a consequence of early sleep disturbances [19,21]; therefore, patients with ADHD are of special interest when examining sleep regulation difficulties. Additionally, dysfunctions in the circadian rhythm may also be involved in the etiology [3,8,9,22,23] of sleep and ADHD, leading to a shift in circadian timing that results in a long sleep onset latency [24,25]. A recently published case–control study of 60 medication-naïve ADHD patients and 60 controls aged 6–16 years analyzed the daily profile of motor activity using actigraphs and found a tendency towards a late chronotype in ADHD [26]. Patients with ADHD may therefore experience a lack of sleep during the week due to social conventions being tailored to the early chronotype (like going to school or to work, which requires getting up early) and try to compensate for this on the weekends. The differentiation between days of the week (school day vs. free day) is therefore of crucial interest in order to detect specific sleep disturbances that may be linked to circadian timing delays.

A meta-analysis examining objective sleep assessments in ADHD patients (actigraphy/polysomnography) only confirmed a lighter sleep (derived from polysomnography assessment; more stage-1 sleep), a tendency for a longer sleep onset latency, and a shorter total sleep time as derived from actigraphy measures [4]. Additionally, a systematic review of sleep and circadian rhythms in children with ADHD aged 5–13 years [27], including both objective (e.g., polysomnography, actigraphy) and subjective measures (e.g., sleep diaries/questionnaires), highlighted heterogeneous results in sleep and ADHD. The findings included shorter sleep duration, longer sleep onset latency, lower sleep efficiency, greater wakening after sleep onset, and sleep fragmentation in ADHD, based on objective sleep measures [28,29,30]. A study examining 8th graders (mean age of 13 years) with (*n* = 162) and without ADHD (*n* = 140) found shorter actigraphy school night sleep duration, more adolescent- and parent-reported daytime sleepiness, more parent-reported difficulties initiating and maintaining sleep, and more total sleep disturbances in the adolescents with ADHD [31]. This contradicts other studies that reported no differences in sleep between children with ADHD and typically developing children [32,33,34]. The reviewed studies have been criticized for the rather restricted focus on the mean of the examined sleep variables, thereby neglecting intra-individual variability (IIV) of sleep parameters [35] that have been shown to be altered in adults with ADHD [36,37]. Findings on IIV in sleep variables in children and adolescents with ADHD are mixed, with some studies reporting greater IIV in sleep duration and sleep onset latency [35,38,39] and others finding no such differences [40,41]. Further contributors to the heterogeneity of previous findings may lie in the various different definitions and methods of scoring of actigraphy variables/data, a failure to consider differences between specific ADHD subtypes, and a varying degree of severity of ADHD symptoms [27,42]. Nevertheless, the aforementioned systematic review concluded that sleep disturbances in children with ADHD are common and may worsen behavioral outcomes, leading to cognitive and emotional impairments [27]. This underlines the relevance of further research on sleep alterations in ADHD.

In general, sleep problems (especially short sleep duration) are associated with various negative consequences for attention, especially regarding academic and cognitive performance, as well as for mental and physical health [43,44,45,46], both of which are already impaired in children with ADHD [47,48]. Even moderate sleep restriction can lead to poorer neurocognitive functioning in children with ADHD and in healthy controls [49], as well as to sleepiness, inattention, and oppositional behavior in adolescents with ADHD [50]. In particular, in light of findings that sleep problems may account for deficits in neurocognitive function (e.g., inhibition performance) over and above ADHD symptom severity, it is crucial to focus on sleep behavior in persons with ADHD [51]. Interestingly, on a neurophysiological level, sleep-deprived drowsiness is associated with slow theta activity derived from electroencephalogram (EEG) during resting state [52,53]. Linking EEG markers (especially theta activity during rest) to sleep problems in children with ADHD might enable a better understanding of the associated and underlying neurobiological mechanisms. The loss of validity of the theta–beta ratio as a diagnostic marker of ADHD over the last 20 years, for example, might in turn be linked to an overall reduction of sleep duration in the general population of children as one conceivable explanation [54]. EEG anomalies, and especially altered slow wave EEG rhythms, are common in children with ADHD and can reflect increased tiredness but are also linked to normal development [55,56,57,58]. This highlights the transdiagnostic relevance of the circadian rhythm and the arousal system (c.f. Research Domain Criteria (RDoC) of the National Institute of Mental Health) and emphasizes the need for future research. While earlier studies identified specific EEG markers related to ADHD [59,60,61], the results are still heterogeneous [62] and no sleep specific markers in wake EEG have been found to date.

To extend the existing heterogeneous literature on ADHD and sleep and improve the current knowledge, this study focused on children with ADHD, with only the combined or hyperactive/impulsive presentation, and compared them to typically developing children aged 6–12 years. Actigraphy data were collected for a duration of 14 days to ensure that enough free days and school days were included for analysis using linear mixed models. Furthermore, accompanying sleep diaries were implemented to validate actigraphy data and control for artifacts (e.g., sleeping in a car) and non-wearing times. It was hypothesized that children with ADHD would show more sleep problems (longer sleep onset latency, lower sleep efficiency, and more sleep deficits) and a higher IIV with regard to their sleep parameters compared with controls. For these parameters, we expected larger group differences with more sleep problems in the ADHD group on school days compared with free days. Further, we assumed later chronotypes and, consequently, later sleep onset times in the ADHD group compared to controls. Moreover, we hypothesized that children with ADHD would show more sleep problems early in infancy (retrospectively assessed using a questionnaire) compared with controls, which would predict later sleep problems. An association between sleep parameters (beyond ADHD symptoms) and neuropsychological functioning (assessed with a cued continuous performance task (CPT)) was assumed, with worse sleep being associated with deficits in CPT performance (more commission/omission errors, longer reaction time, and higher reaction time variability).

## 2. Materials and Methods

### 2.1. Participants

Participants were *n* = 57 children (*n* = 15 females; age in years: 6–12 at inclusion in the ESCAschool study; M = 9.38, SD = 1.77), consisting of *n* = 24 children with ADHD (4 females; age in years: M = 9.66, SD = 1.95) and *n* = 33 healthy control children (11 females; age in years: M = 9.18, SD = 1.64). In the group of children with ADHD, *n* = 20 were medicated with stimulants (*n* = 19) or atomoxetine (*n* = 1). Participants were recruited from the sample for the ESCAschool multicenter trial. ESCAschool investigates stepped-care treatments for school-aged children with ADHD, within the framework of the ESCAlife (Evidence-based, Stepped Care of ADHD along the life span) consortium. The study protocol and data acquisition for ESCAschool have been published previously [63] and were registered in the German Clinical Trials Register (reference number: DRKS00008973 at https://www.drks.de/drks_web/, accessed on 3 September 2021). Sleep assessment with actigraphy and recruitment of the participants were only conducted at the Mannheim center. Ethical approval was granted by the local ethics committees and written informed assent/consent was obtained from the participating children and their parents/legal representatives. Exclusion criteria were IQ < 80 and insufficient German language and reading skills of parents/legal representatives with respect to relevant assessments (e.g., filling out questionnaires) and therapeutic interventions. Furthermore, children with a diagnosis of pervasive developmental disorder, schizophrenia, bipolar disorder, severe depressive episodes, epilepsy, heart disease, and current or planned intensive behavioral therapy for ADHD or oppositional behavior on a weekly basis were excluded. As the protocol for the ESCAschool study also included pharmacotherapy, further exclusion criteria were a known non-response to standard ADHD medication (methylphenidate, (lis)dexamphetamine, and atomoxetine), psychotropic medication (other than for ADHD), or neuroleptic medication (other than for the treatment of disturbance of impulse control).

### 2.2. ADHD Diagnosis and Comorbidities

ADHD diagnosis was assessed using the clinician-rated ADHD Checklist (Diagnostic System for Mental Disorders in Children and Adolescents (DISYPS-III [64]), Diagnose-Checklist ADHS (DCL–ADHS–clinical interview), and based on parent interview prior to any treatment or measurement, in a medication-free status of the child. This checklist reflects the symptoms of ADHD according to the diagnostic criteria of the DSM-5 and the *International Classification of Diseases* 10th edition (ICD-10) and is recommended in the German guidelines [65] on assessment and treatment of ADHD. Furthermore, the parents and—if consent was obtained—the teacher completed clinical questionnaires regarding ADHD symptoms, within the same diagnostic system described above (‘Fremdbeurteilungsbogen ADHS’ (FBB–ADHS–Parent–Teacher)) in order to include various settings and perspectives on the child’s symptoms during the diagnostic process. All children were screened for comorbidities by experienced clinical psychologists using standardized clinical interviews (DCL-screened comorbidities, DISYPS-III) in order to rule out that ADHD symptoms were caused by another psychiatric disorder (e.g., depression, anxiety).

### 2.3. Sleep Measures and Chronotype

Actigraphy: Sleep was recorded using actigraphs (ActiGraph LLC, Pensacola, FL, USA, model: wGT3X-BT) and were intended to be worn on the non-dominant wrist for 14 days and nights (24 h) to ensure that four free days and five school days [66] were included in the actigraphic assessment for subsequent multilevel modeling. Participating children were instructed to take off the actigraph only for showering, bathing, and sports. The ActiLife software (version 6.13.4) was used to download the data in sixty-second epochs. Sleep scores were calculated using the Sadeh sleep scoring algorithm [67]. Actigraphy measures were accompanied by daily diaries, which were completed by parents to validate bedtimes and non-wearing times of the actigraphs. The following actigraph variables were recorded: sleep onset latency (SOL—time between the child going to bed and falling asleep), sleep efficiency (number of sleep minutes divided by the total number of minutes the child was in bed), total counts (TC—the total actigraphy counts (movements) summed up for the entire sleep period), sleep deficit (calculated from the recommended age-dependent sleep duration [68], minus the total sleep time (TST)), and sleep onset time (SOT—the first minute that the algorithm scores ‘asleep’). A special focus was on the sleep variables rather than the wake epochs due to the better sensitivity (epochs scored as sleep, such as TST) of the actigraphs compared to the lower specificity (epochs scored as wake, e.g., wake after sleep onset) [42].

*Sleep in infancy*: A short version of the Revised Dimensions of Temperament Survey (DOTS-R; [69]) questionnaire was used to assess sleep problems at toddler age (18–36 months), using one item (‘the child had difficulties falling and staying asleep’).

*Chronotype*: We used the Munich ChronoType Questionnaire (MCTQ [14]) for children (MSFsc—midsleep on free days, corrected for oversleeping) to assess the chronotype, which is a good predictor of DLMO [16].

### 2.4. Neuropsychological Performance

The cued continuous performance task (CPT) is a standardized computer-administered test. Single letters are presented sequentially for a total duration of approximately eleven minutes. The participant should press a button if an ‘X’ follows an ‘O’. The measures of interest derived from the CPT are omission errors (missed targets), commission errors (false hits), percentage of hits, reaction time (RT), and RT variability (RTV). The CPT task was performed in the morning of a school day during the diagnostic phase of the study around, three months before wearing the actigraph.

### 2.5. Statistical Analyses

Group differences regarding age, IQ, sex, and the number of days on which the actigraph was worn (including differentiation between free days and school days) were analyzed with IBM SPSS (version 27).

To explore group differences in sleep variables, a random-intercept, mixed-effects model (accounting for intra-individual clustering of observations), using R version 4.0.4 (package: lmer-Test, Version 3.1–3), was applied for each actigraphy variable separately as dependent variable (SOL, sleep efficiency, TC, sleep deficit, and SOT). As fixed factors, the group (ADHD or control) and day of the week (free day or school day) were specified in order to detect potential differences between free days and school days. In a second step, sleep problems in infancy were added as a fixed factor to analyze the predictive value for later sleep problems in childhood. Subsequently, the standard deviation (SD), as a value for intra-individual variability (IIV) for free days and for school days, was calculated separately for each participant and each actigraphy variable. The resulting data were analyzed by means of the mixed model with IIV—free/IIV—school as the dependent variable and group as a fixed factor. Additionally, *t*-tests were calculated in a post hoc analysis. To find group differences in the chronotype (MSFsc), independent samples *t*-tests were calculated.

To investigate the potential additional effect of current sleep problems, beyond age and ADHD symptoms, on CPT performance, we applied a stepwise regression model. Results regarding neurophysiological measures can be found in Appendix A.2. Statistical significance was set at *p* < 0.05. Effect sizes were low with partial η² = 0.01, medium with partial η² = 0.06, or large with partial η² = 0.14.

## 3. Results

### 3.1. Participiants’ Characteristics

Demographic information and group differences between ADHD patients and controls can be found in Table 1. To explore effects of medication in our cohort, we analyzed effects of the dosage of ADHD medication in patients on ADHD symptom severity and sleep variables. Correlational analyses revealed no significant association with ADHD symptom severity or any of the sleep variables when controlled for age (*p* > 0.05). All medicated ADHD children received their medication in the morning. Four children had an additional dosage of an unretarded methylphenidate at noon. The dosage of methylphenidate ranged from 15 mg/d to 60 mg/d (50% of the medicated ADHD children received ≤20 mg; 75% ≤ 30 mg/d). Melatonin was used in *n* = 3 children with ADHD (sensitivity analysis was conducted).

### 3.2. Actigraphy Sleep Results

#### 3.2.1. Mean Analyses of Actigraphy-Derived Sleep Variables

The descriptive statistics of the sleep variables are presented in Table 2. The median of the total wearing time for the actigraph was 14 days (range: 7–21 days) for children with ADHD and 15 days (range: 10–22 days) for the controls, and did not differ significantly between the groups. The number of school days (ADHD median: 9 days; controls median: 8 days) or free days (ADHD median: 6 days; controls median: 7 days) on which the actigraphs were worn also did not differ significantly between children with ADHD and controls. In-bed time and out-of-bed time were nearly the same in the two groups, regardless of the day of the week.

**Figure 1:**
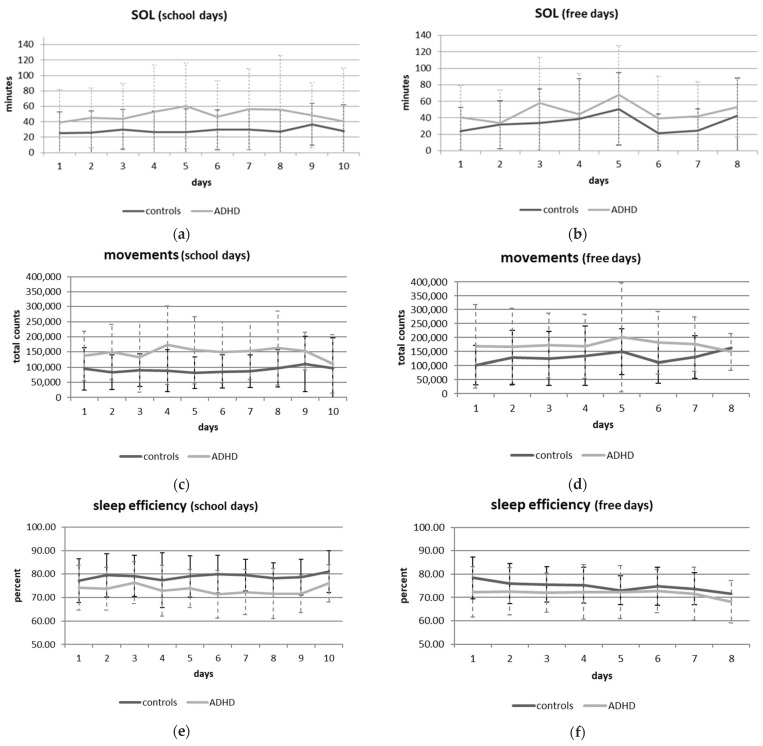

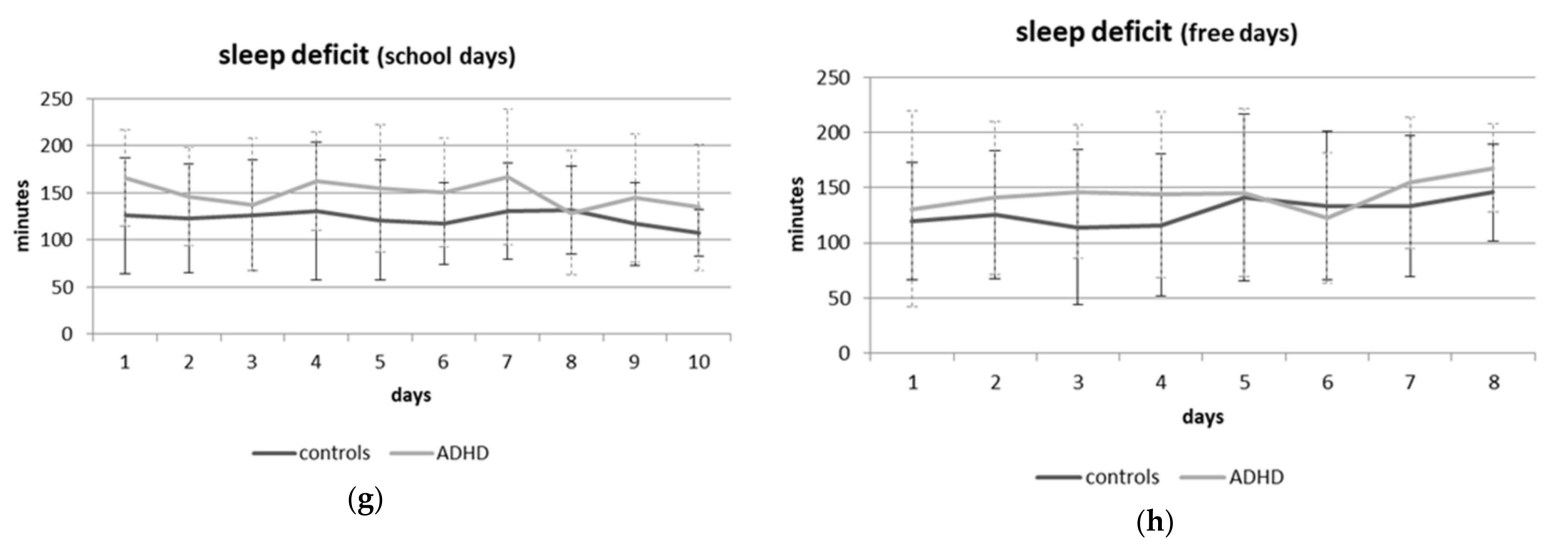
Group differences in SOL: on school days (**a**) and free days (**b**); in total number of movements on school days (**c**) and free days (**d**); in sleep efficiency on school days (**e**) and free days (**f**); and in sleep deficit on school days (**g**) and free days (**h**).

Group differences in sleep variables: Mixed effects models were fitted to the data, with each sleep variable separately as dependent variable, controlling only for IQ and sex as groups were age-matched (fixed factors: group, day of week, group x day of week). We found significant main effects of group for SOL (t (67) = 2.817, *p* = 0.006, partial η² = 0.11, Figure 1a,b), TC (t (59)= 2.718, *p* = 0.009, partial η² = 0.11, Figure 1c,d), and sleep efficiency (t (60) = −2.128, *p* = 0.037, partial η² = 0.07, see Figure 1e,f) with medium to large effect sizes. Furthermore, day of the week was a significant factor in TC (t (826) = 3.575, *p* < 0.011) and for sleep efficiency (t (831) = −2.554, *p* = 0.011). We observed trend level effects of group on sleep deficit (t (64) = 1.864, *p* = 0.067, see Figure 1g,h) and also a trend-level interaction of group x day of week on sleep deficit (t (829) = −1.927, *p* = 0.054). The results did not change substantially when age was included in mixed-effects models as a fixed factor, with the exception of sleep deficit (t (63) = 2.027, *p* = 0.047). Post hoc analyses with independent samples *t*-tests revealed that children with ADHD needed more time to fall asleep (20 min on school days: t (46) = −2.671, *p* = 0.010; 15 min on free days: t (55) = −2.317, *p* = 0.024) than typically developing children. Children without ADHD moved less during sleep than children with ADHD did, as assessed by TC, regardless of the day of the week (school days: t (46) = −3.346, *p* = 0.002; free days: t (55) = −2.598, *p* = 0.012). Moreover, sleep efficiency was lower in children with ADHD (minus 5%) regardless of the day of the week (school days: t (46) = 2.218, *p* = 0.032; free days: t (55) = 2.248, *p* = 0.029). The extent of the sleep deficit only differed between the groups on school days (t (46) = −2.135, *p* = 0.038), with ADHD children experiencing more sleep deficits on school days compared with typically developing children. No group differences emerged on free days (t (55) = −1.188, *p* = 0.240). Paired samples *t*-tests revealed that on free days, all children moved more during sleep (TC: t (47) = −3.757, *p* < 0.001) and had lower sleep efficiency (t (47) = 2.335, *p* = 0.024), but no differences in sleep deficit were found (t (47) = 1.089, *p* > 0.05). Only marginally significant effects were found regarding a group x day of week interaction effect on sleep deficit. Children with ADHD tend to experience a greater sleep deficit on school days compared with free days; whereas, children without ADHD tend to experience greater sleep deficit during free days. When only considering children with a sleep duration of less than 7 h [68], which is the minimum of the recommended sleep length for this age group (mean recommended 9–11 h [68]), 32% of children with ADHD fell below this cut-off on school days, whereas only 12% of control children did so. Similar differences emerged on free days, with 29% of children with ADHD and 6% of control children sleeping less than 7 h per night.

*Sleep problems in infancy*: Adding sleep problems in infancy as an additional fixed factor in a mixed effects model and controlling for IQ and sex, we found a significant large effect of sleep deviations in infancy on SOL (t (51) = 3.114, *p* = 0.003, partial η² = 0.16) and a medium effect on TC (t (50) = 2.716, *p* = 0.009, partial η² = 0.13), but no significant effect on sleep efficiency (t (50) = −1.086, *p* > 0.05).

#### 3.2.2. Analyses of Variability (IIV) of Sleep Variables

The mixed effects models for IIV for sleep variables (fixed factor: group, day of week, group x day of week), controlling for IQ and sex, revealed main effects of group only for SOL (t (79) = 2.487, *p* = 0.015, partial η² = 0.07) with a medium effect size (Figure 1a,b) and a trend-level group effect for SOT (t (95) = 1.748, *p* = 0.084). Main effects for day of week were found on TC (t (52) = −2.874, *p* = 0.006), sleep efficiency (t (99) = −2.610, *p* = 0.011), and sleep deficit (t (55) = −3.647, *p* = 0.001), regardless of group. Post hoc analyses with independent samples *t*-tests showed higher IIV in SOL in children with ADHD than controls, regardless of the day of the week (school days: t (46) = −3.084, *p* = 0.003; free days: t (55) = −2.322, *p* = 0.024). IIV in SOT was only higher in children with ADHD compared with controls on school days (t (46) = −3.601, *p* = 0.001). In general, we found greater IIV in TC, sleep efficiency, and sleep deficit on free days for children both with and without ADHD, as indicated by paired samples *t*-tests (sleep efficiency: t (47) = 2.251, *p* = 0.029; sleep deficit: t (47) = 3.123, *p* = 0.003; TC: t (47) = −2.866, *p* = 0.006).

### 3.3. Chronotype and Sleep Onset Time

**Figure 2:**
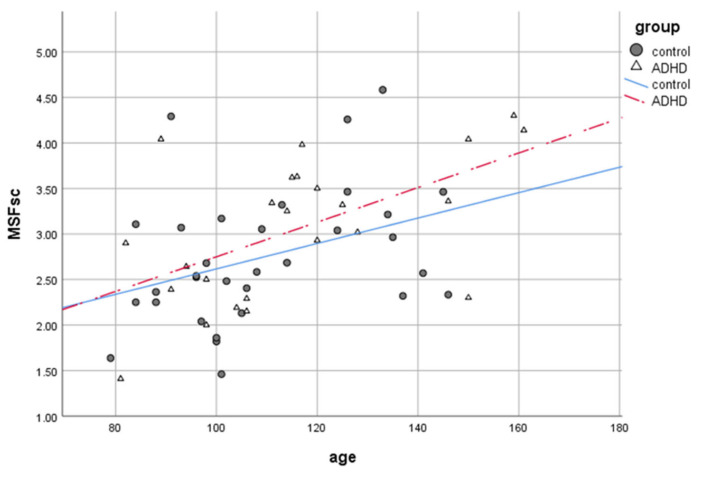
Chronotype (MSFsc: lower scores reflect earlier chronotypes) changes with age (in months) by group.

Independent samples *t*-tests revealed no differences in chronotype between groups (ADHD or control; t (54) = −1.481, *p* > 0.05), except from age effects (Figure 2). The SOT, which could also be a relevant marker for circadian timing, did not differ between ADHD and control children. Analyses with a mixed model only found main effects for day of the week (*p* < 0.001) and sex (*p* = 0.026). Post hoc analyses with paired-samples *t*-tests revealed that both ADHD and control children had a later SOT on free days than on school days (t (47) = −9.894, *p* < 0.001), with females falling asleep earlier than males (school days females/males: 8:53 p.m./9:33 p.m.; free days females/males: 9:46 p.m./10:21 p.m.).

### 3.4. Effects on Neuropsychological Performance

Regarding CPT measures, *n* = 44 children (*n* = 13 ADHD, *n* = 31 controls) completed the task and were included in the analysis. A stepwise multiple regression model was fitted to explore whether age (step 1), ADHD symptoms (step 2), and sleep variables on school days (step 3) or free days (step 4) predicted CPT outcome. At step 1, age contributed significantly to reaction time of hits (F (1,33) = 9.012, *p* = 0.005) and accounted for 21.5% of the variance. The ADHD symptom score at step 2 contributed significantly to the regression model for omission errors (F (1,32) = 17.222, *p* < 0.001) accounting for 34.9% of the variance. Due to Bonferroni correction (*p* < 0.005) reaction time variability of hits (F (1,32) = 5.195, *p* = 0.029) and commission errors (F (1,32) = 8.438, *p* = 0.007) were only marginally significant, accounting for 12.6% of the variance in reaction time variability and 20.2% of the variance in commission errors. Step 4 revealed only a trend for sleep variables on free days (SOL, TC, sleep efficiency, and sleep deficit) for commission errors (F (1,24) = 2.416, *p* = 0.077). No further significant effects were found on any of the sleep variables on school days for CPT outcome beyond the ADHD symptom score. Sleep and demographic data of the subgroup of ADHD and control children that completed the CPT task are listed in Appendix A.1.

## 4. Discussion

The main objective of this study was to explore sleep problems in children with ADHD compared to their typically developing peers, using extended actigraphy and mixed models, with a particular focus on IIV in the sleep variables. Additionally, we analyzed precursor symptoms and the chronotype in order to detect possible early risk factors for current sleep disturbances and to clarify the potential occurrence of a circadian phase delay in this age group. Furthermore, effects of sleep (problems) on neuropsychological functioning and associations with EEG markers were explored.

The results show that, in our sample, children with ADHD experienced more sleep problems (longer SOL, lower sleep efficiency, more movements during sleep) than typically developing controls on school days and on free days, but no significant differences were found in chronotype or sleep onset time. The IIV varied with regard to SOL (higher IIV in children with ADHD), independently, of the day of the week. Only the extent of sleep deficit was slightly higher in children with ADHD on school days than on free days as compared with controls. These findings are in line with the results of a recent meta-analysis [4], which revealed longer SOL and lower sleep efficiency, and with a systematic review on school-aged children with ADHD [27], which confirmed the occurrence of sleep disturbances using subjective and objective measurement methods in ADHD. With regard to IIV, children with ADHD showed higher IIV than typically developing children only in the SOL, which confirms the results of some earlier studies [39,70], but also contradicts others [40,41] that did not find greater IIV in children with ADHD. Environmental factors, such as having a regular bedtime routine implemented by the parents or caregivers and no screen time or light exposure before going to bed [71], are influencing variables that may result in later or more variable sleep onset times. These structural hints require more attention—especially in ADHD families—since it was shown that parents are more negligent in implementing their children’s bedtime routines on free days compared with school days [72]. This may affect the circadian rhythm and contribute to prolonged sleep onset in general. On the other hand, sleep hygiene aspects and parenting domains like consistency or warmth represent important modifiable factors in the management of sleep problems in children with ADHD [72]. A further influencing factor for prolonged or variable sleep onset time that has often been discussed in the literature is a delay in circadian timing, resulting in a later chronotype in children with ADHD, which could not be confirmed in our sample. However, we used the MCTQ, which does not reflect the child’s sleep time preference (in contrast to the Morningness–Eveningness Questionnaire, MEQ [73]), but rather the actual bedtime, which is normally determined by the parents. The chronotype data reflect the age-dependent component very well, being later with older age (even in this narrow age range) and with male sex, leading to the conclusion that the data are valid despite the relatively small sample size. Moreover, they also highlight the influence of age on sleep variables (especially the chronotype) in this age group, when substantial changes occur. Additionally, since most of the children with ADHD were medicated, an influence of psychopharmacological treatment on circadian timing or sleep in general cannot be precluded [74]. Hypotheses of altered dopaminergic functioning related to circadian rhythm disturbances have been proposed, which may contribute to ADHD symptoms and etiology [22]. However, effects of ADHD medication on sleep are still unclear, with some studies reporting more sleep disturbances under ADHD medication [75,76] and others unable to find a negative influence on sleep [77], or even reporting an alleviating effect on sleep behavior [78,79]. An additional dose of a short-acting stimulant is sometimes recommended in the afternoon or before bedtime in order to counteract the rebound effect [80,81]. Prescribing information for stimulant medications, such as methylphenidate, nevertheless lists insomnia as a common adverse reaction [82]. Screening for sleep problems before starting on a stimulant medication is important and highly recommended, since sleep problems may already exist before taking the medication, which may aggravate the sleep (onset) problems. Additionally, the interaction of dopamine and circadian rhythms have not yet been conclusively clarified. Previous studies already tried to disentangle medication from ADHD effects and found no differences in parent reported sleep problems while their child received a psychostimulant but even recognized a stabilizing effect of psychostimulants on sleep (IIV of SOL of children using a psychostimulant did not differ from the control group) [39]. Further, it was shown that there were no significant differences in sleep between the medicated and unmedicated state of the children while receiving methylphenidate [83].

A further finding of this study is the predictive value of sleep problems in infancy for later SOL and the total number of movements (TC) during sleep in childhood, but not for sleep efficiency. These findings are in line with a previous study, investigating the long-term effects of infant sleep problems on the development of ADHD at the age of 5.5 years [21]. Thus, difficulties in falling asleep could be more crucial than sleep problems during the night (e.g., increased awakening) and may result in a shortened total sleep time. Possible explanations for the link between regulatory problems and behavioral problems in childhood include early care-giving relationships, the infant’s temperament, and disturbances in the dopaminergic system [19,84,85]. Interestingly, dopamine is not only involved in reward processes and motivation but is also an important regulator of central and peripheral circadian rhythms [86,87,88]. This highlights the overlap between sleep regulation processes and neurodevelopmental disorders, such as ADHD, and emphasizes the transdiagnostic approach of sleep/wake rhythms and the arousal system. However, due to the restricted validity of the assessment of sleep problems in infancy (with only one item being assessed retrospectively) the predictive value needs to be explored in future studies, preferably with longitudinal designs and more comprehensive methods.

Looking at the potential impact of sleep on neurocognitive functioning and the association with EEG biomarkers, the present study did not identify any significant effects after controlling for age. This illustrates the substantial influence of age on sleep variables, which should especially be focused on in the transition from childhood to adolescence. Nevertheless, a tendency emerged for impulsive control deficits (more commission errors) being associated with sleep disturbances on free days, beyond the effect of ADHD symptoms and age, which is in line with previous findings [51]. Additionally, the ADHD group in our sample were medicated while the EEG was recorded. Treatment with stimulant medication can have a normalizing effect on EEG patterns in children with ADHD [89]. Moreover, a review demonstrated an increase in theta activity due to fatigue and shortened sleep duration [52], which is one of the most common findings in EEG patterns in children with ADHD [61], thus further underlining the close relationship between markers characteristic for ADHD and sleep disturbances. Additionally, it provides further support for the suggestion that at least a subgroup of children with ADHD may suffer from hypoarousal [83,90]. This suggestion is consistent with the EEG vigilance model [91] which describes the transition from relaxed wakefulness through drowsiness to sleep onset in an eyes-closed resting period. An unstable vigilance regulation with a quick drop to lower EEG vigilance stages and characteristic drowsiness EEG patterns (excess theta and/or enhanced theta–beta ratio (TBR)) has been described for patients with ADHD [92].

Nevertheless, for treatment, and for the well-being of the children and their families, it is crucial to not only consider core ADHD symptoms as relevant outcomes to be measured, but also to place a greater focus on accompanying factors such as quality of life (for which sleep problems are highly relevant). In particular, sleep onset problems can have a fundamental impact on the interaction between parents and child and may aggravate existing behavior problems.

The results of this study need to be interpreted in the light of some limitations. First, only children with ADHD hyperactive/impulsive or combined presentation were investigated, which limits the generalizability of the findings. However, sleep problems seem to be predominantly prevalent in these presentations of ADHD [26], which corresponds to our finding of more movements during sleep as a differentiating variable between ADHD and typically developing children. This supports the hypothesis of hypoarousal in at least a subgroup of children with ADHD with compensating hyperactive behavior. Second, our sample size was relatively small, and even smaller for the EEG and CPT assessments. This limits the validity of our findings due to low statistical power. Third, as the children with ADHD were medicated, we are unable to draw conclusions regarding ADHD children without medication. A more balanced sampling of medicated and unmedicated children with ADHD would have been important to disentangle ADHD and medication effects. This was not feasible given the guideline-based, stepped-care design of our multicenter study. However, several previous medication RCT [77,93] found no or only minor effect of medication compared to ADHD status on sleep actigraphy measures, and our exploratory analysis did not reveal a role of medication dosage. Fourth, disturbed sleep in infancy was collected using a single item from a questionnaire with retrospective parent information, which may lead to bias and thus limit the generalizability. There is a need to investigate the predictive value of early regulatory deficits by more sophisticated methods using, for example, more comprehensive assessments and longitudinal designs to draw further conclusions. However, this study investigated a relatively homogenous group of children with ADHD, who wore the actigraph for a longer time period than most of the earlier studies, which were often criticized, as a minimum of five nights is recommended in order to achieve valid actigraphy data [66]. Considering the aforementioned differences in circadian timing depending on the day of the week, five days are not sufficient to draw conclusions on sleep behavior for free days and school days separately. Therefore, the long wearing time of the actigraph can be regarded as a strength of this study, also with regard to IIV, as it enabled clustered observations and separate analyses for school days and for free days using linear mixed models.

Future studies with larger sample sizes, including both medicated and unmedicated children with ADHD with a narrow age range, are needed to clarify the described interaction between sleep problems and ADHD symptoms. There is a need to investigate the underlying mechanisms of sleep and ADHD—ideally by identifying (neuro-) biological markers and placing a greater focus on IIV in sleep variables. Special attention should be paid to early regulatory problems as a risk factor for later behavior problems, by exploring neurofunctional and genetic mechanisms in order to implement preventive strategies in infancy. Investigating the activity profile of ADHD children and healthy controls over 24 h could help to better understand the circadian rhythms in both groups and to find individual treatment strategies for (sleep-deprived) children with ADHD.

## 5. Conclusions

The present findings are in line with the results of earlier studies on sleep and ADHD and extend previous knowledge by addressing IIV using linear mixed models. We found disturbances in several sleep parameters, with a strength of the study being the long wearing time of the actigraphs and a limitation being the relatively small sample size. The predictive value of early sleep problems for later sleep onset problems provides further information for future studies that should investigate the importance of early sleep problems in longitudinal designs. This can pave the way for new therapeutic approaches and emphasizes the importance of early intervention strategies. The need to assess sleep behavior/deficits in routine clinical care, especially in children with ADHD with combined and hyperactive/impulsive presentations, is important. This could help improving core ADHD symptoms as well as the quality of life of children with ADHD and their families and should be tested in future studies.

## Figures and Tables

**Table 1:** Demographic information.

	ADHD *n* = 24	Controls *n* = 33	Test Statistic	*p*
Age (SD)	9.66 (1.95)	9.18 (1.64)	F ₁‚₅₅ = 0.182	0.32
Sex (m/f)	20/4	22/11	X² (1) = 12.789	<0.001 ^1^
IQ (SD)	106 (14.7)	118 (10.8)	F ₁,₅₅ = 3.389	<0.001 ^1^
ADHD symptom score	2.16	0.20	F ₁,₅₅ = 2.670	<0.001 ^1^
Combined subtype	20/24	/		
Hyp/Imp. subtype	4/24	/		
Comorbid ODD	11/24	/		

^1^ The groups differed significantly regarding sex and IQ as well as regarding ADHD-specific markers.

**Table 2:** Descriptive statistics of sleep variables, means reported. SD—standard deviation.

Sleep Variables	ADHD	Controls
Sleep Onset Latency (SD)		
school days	49 min (33 min)	29 min (18 min)
free days	46 min (29 min)	31 min (21 min)
Total Counts (SD)		
school days	151,214 (76,962)	91,198 (45,602)
free days	182,795 (113,454)	121,787 (62,562)
Sleep Efficiency (SD)		
school days	74% (7%)	79% (7%)
free days	72% (8%)	76% (6%)
Sleep Deficit (SD)	+/−2 h Normal	+/−2 h Normal
school days	2 h 32 min (42 min)	2 h 3 min (49 min)
free days	2 h 22 min (56 min)	2 h 6 min (44 min)
Total Sleep Time (SD)		
school days	7 h 25 min (54 min)	7 h 58 min (48 min)
free days	7 h 37 min (53 min)	7 h 56 min (42 min)
Sleep Onset Time (SD)		
school days	9:27 p.m. (46 min)	9:20 p.m. (48 min)
free days	10:19 p.m. (52 min)	10:06 p.m. (57 min)

## Data Availability

Due to ethical, legal or privacy issues, data should not be shared.

## Appendix A

### Appendix A.1. Sleep and Demographic Data of the Subgroup of ADHD and Control Children That Completed the CPT Task

**Table A1:** Demographic information of the subgroup that completed the CPT task.

	ADHD *n* = 13	Controls *n* = 31	Test Statistic	*p*
Age (SD)	8.78 (1.56)	9.07 (1.60)	F ₁‚₄₂ = 0.213	0.594
Sex (m/f)	10/3	21/10	X² (1) = 7.364	0.007
IQ (SD)	103 (11.8)	118 (11.0)	F ₁,₄₂ = 0.820	<0.001
ADHD symptom score	2.24	0.20	F ₁,₄₂ = 0.304	<0.001
Combined subtype	11/13	/		
Hyp./Imp. subtype	2/13	/		
Comorbid ODD	4/13	/		

**Table A2:** Descriptive statistics of sleep variables of the subgroup that completed the CPT task, means reported. SD: standard deviation.

Sleep Variables	ADHD (*n* = 13)	Controls (*n* = 31)
Sleep Onset Latency (SD)		
school days	55 min (40 min)	28 min (17 min)
free days	55 min (33 min)	31 min (21 min)
Total Counts (SD)		
school days	184,058 (87,277)	91,852 (46,775)
free days	220,946 (138,619)	121,377 (62,223)
Sleep Efficiency (SD)		
school days	74% (8%)	78% (8%)
free days	70% (10%)	76% (6%)
Sleep Deficit (SD)	+/−2 h Normal	+/−2 h Normal
school days	2 h 31 min (52 min)	2 h 7 min (49 min)
free days	2 h 38 min (63 min)	2 h 8 min (43 min)
Total Sleep Time (SD)		
school days	7 h 37 min (64 min)	7 h 57 min (49 min)
free days	7 h 33 min (65 min)	7 h 56 min (39 min)
Sleep Onset Time (SD)		
school days	9:18 p.m. (53 min)	9:20 p.m. (49 min)
free days	10:00 p.m. (57 min)	10:04 p.m. (55 min)

The analysis of the main results of sleep variables between the groups in the subgroup sample of ADHD and control children, who completed the CPT task, revealed the same significant main effects of group for SOL (t (51) = 2.644, *p* = 0.011, partial η² = 0.12) and TC (t (45) = 2.719, *p* = 0.009, partial η² = 0.14). Sleep efficiency and sleep deficit were not significant (*p* > 0.05). Adding sleep problems in infancy as an additional fixed factor in the mixed effects model, we found the same significant large effect of sleep deviations in infancy on SOL (t (38) = 3.101, *p* = 0.004, partial η² = 0.20) and on TC (t (37) = 2.815, *p* = 0.008, partial η² = 0.18) in the subgroup. Summarizing the results, we conclude that the subgroup that completed the CPT task is representative for the total ADHD/control group.

### Appendix A.2. Associations of Sleep Variables with EEG Parameters

*Hypothesis*: Regarding the relationship between sleep parameters and selected EEG indices, we expected to find negative correlations between theta oscillations during resting state and sleep onset latency/later chronotype.

*Material and methods*: The EEG was recorded using THERAPRAX full-band DC-EEG amplifier systems (with a high input impedance >10 GΩ for proprietary impedance control; neuroCare GmbH, Germany) during resting state (first with eyes open and then with eyes closed, each for 4 min) and during the CPT. The resting-state EEG and the EEG during the CPT were recorded using a 22-channel EEG cap (Brain Products, Gilching, Germany), with a sampling rate of 256 Hz (DC-70 Hz) for the resting-state EEG and a higher sampling rate of 512 Hz during the CPT. Impedances were kept below 20 kΩ. The EEG was recorded in the morning but also until early afternoon (for control children as well as for children with ADHD) of a school day directly before wearing the actigraph.

*Statistical analysis*: Chronotype (MSFsc)/sleep onset problems (SOL) were correlated with slow wave oscillations during EEG at rest and with event-related potentials (ERPs) during the CPT when controlling for age.

*Results*: EEG data were obtained from *n* = 41 children in total (*n* = 8 ADHD children and *n* = 33 controls). Groups were not analyzed separately due to the small sample size of the ADHD group. Partial correlations between EEG frequency band power during eyes open and eyes closed as well as event-related potentials (ERPs: CNV amplitude, Cue P300 latency/amplitude) with sleep variables revealed no significant effects when controlling for age (*p* > 0.05). Only negative correlations with age and EEG parameters were found, especially for slow wave resting frequencies (theta and delta), indicating typical maturational effects. Correlations between theta frequency band power (as a potential marker for drowsiness) and chronotype (lower scores for earlier chronotypes) were significant (r = −0.329, *p* = 0.038), but also became nonsignificant when controlling for age (r = −0.217, *p* > 0.05), Figure 2 (as age was correlated with chronotype scores).
